# The Story of the Insane from Year to Year

**Published:** 1896-09-05

**Authors:** 


### THE STORY OF THE INSANE FROM YEAR TO YEAR.

Ninth Series.

### SUNDERLAND BOROUGH ASYLUM.

This asylum was opened on May 25th, 1895, and a large number of patients chargeable to the Borough, but boarded out in other asylums, were transferred to it. In addition to these transfers, 58 new cases were admitted before the close of the year. On the last day of December there were in residence 288 patients. There were 15 recoveries; 4 discharged relieved, and 13 deaths. The recoveries stand at 25·8 per cent. on the admissions, and the deaths at 4·9 per cent. on the average number resident. Eleven of the deaths were caused by cerebral and spinal diseases. When speaking of the causes of insanity, Dr. Elkins says that such investigations must always be difficult, and that a summary must be imperfect. He does not believe much in moral causes being of themselves sufficient without some inherent brain instability; and he reminds us that Job did not become insane. No. But then Job was a bit of a philosopher, and inclined to take good and bad things easily, up to a certain point at all events. The long sermons his friends preached to him would have tried most other men, however. Although the asylum is brand new, it has already been visited by erysipelas, septic pneumonia, and sore throat, and we are surprised to learn that some of the drains were faulty; but whether the architect or the contractor is to be blamed for this we do not know. Instruction is being given to the attendants and nurses to prepare them for the examinations of the Medico-Psychological Association. Dr. Elkins does not think much of the hospital-"trained" nurse for asylum work. "There are, of course, grand exceptions, but the average trained nurse is a useless person in an asylum, and she is in the 'Mother Gamp' stage of ignorance as to treatment of mental cases.' This agrees entirely with our own experience. The committee say they "have to record their high appreciation of the conduct of the Medical Superintendent, and specially commend him for his arduous exertions in the discharge of the duties of his office in the opening year of the institution." This is all very well; but we do not learn that the committee have formulated a satisfactory pensions scheme yet, or even rescinded their former resolution. The weekly rate is 12s. per head.

### BERKS COUNTY ASYLUM AT MOULSFORD.

There were 554 patients in residence at the beginning of the year, and 558 at the end of the year. There were 89 first admissions, 20 re-admissions, 42 recoveries, 9 discharged relieved, and 48 deaths. Calculated on the admissions, the recoveries stand at 35·6 per cent., and the deaths at 8·6 per cent. on the average number resident. Sixteen of the deaths were caused by cerebral and spinal diseases, 1 by enteritis, 1 by enteric fever, and 8 by consumption. Hereditary influence alone or in combination with other causes accounts for 47 of the year's admissions, and intemperance for 4. Dr. Murdoch thinks he may possibly have 19 curable cases out of the 558 remaining under treatment at the end of the year. Like nearly every other county asylum Berkshire finds that helpless cases accumulate in the infirm wards, and that it will shortly be an urgent question how to provide nursing accommodation for these bedridden cases. A strain is being thrown on the asylums by the emptying of the lunatic wards in our workhouses, and this strain is more felt in the infirm wards than in the others. Then it has often happened that additions have been built to our asylums, while it has been found difficult to add to the infirm wards in the right proportion. There was an outbreak of typhoid fever,and altogether ten people were stricken with the disease,but all recovered. Dr.Murdoch has the following sensible remarks on pensions which remarks we commend to the visitors of the Sunderland and Lincoln Asylums: "As an incentive for suitable persons to enter and remain in the service, there could be no better inducement than the fact of being certain of obtaining a pension when they are no longer able to bear the incessant strain of their work. The principle is now being acted upon by County Councils on the recommendations of their asylum sub-committees." We notice that Nurse Clara Hibberd was granted a pension of £26 per annum after 13 years' service. The visitors record "their continued appreciation of the services of Dr. Murdoch." The average cost per head was a fraction under 7s. 6d. a week.

